# Effectiveness of different methods of health education: A comparative assessment in a scientific conference

**DOI:** 10.1186/1471-2458-5-88

**Published:** 2005-08-22

**Authors:** Asim Saha, Era Poddar, Minal Mankad

**Affiliations:** 1 Occupational Medicine Division, National Institute of Occupational Health, Ahmedabad, India; 2 Ergonomics and Work Physiology Division, National Institute of Occupational Health, Ahmedabad, India; 3 Reproductive Toxicology Division, National Institute of Occupational Health, Ahmedabad, India

## Abstract

**Abstract:**

**Background**

Every individual mode of health education has its own merits, drawbacks as well as their own sphere of effectiveness. A specific mode of communication is more useful in a specific setting on a specific group than others. To search for optimum mode of communication for a specific audience is a major area of research in health education. The issue of imparting health education to a gathering of educated people, representing different fields of knowledge has remained a relatively less lighted aspect of health education research. In this backdrop this study was initiated for making a comparative assessment of different methods of dissemination of health education among educated people.

**Methods:**

A cross-sectional interviewer administered questionnaire survey was conducted involving 142 randomly selected subjects during the last session of a five-day conference having health as main theme when the opinion of the delegates regarding different communication methods was asked for. Collected data was analyzed not only to find out the optimum mode of education dissemination in such a setting but also to find the contribution of different factors in the preferences of the study subjects.

**Results:**

The participants opted more (60%) for focused programs of smaller audience (sectional program). In both broad area (main program) and focused area programs (sectional), the participants preferred lectures (62% and 65.7% respectively). Specific topics were preferred both in lectures (67.6%) and symposia (57.7%). In the exhibition, exhibits seemed to be more attractive (62%) than the posters. Qualification has emerged to be a contributing factor in peoples' choice towards sectional programme and also in their affinity to symposia. Increased age was a significant contributor in participants' preference towards specific topics. Physical barriers of communication appeared to be a problem in the main program as well as in the exhibition. Lack of coherence among the speakers was reported (69%) to be a major reason for which symposia was not preferred.

**Conclusion:**

This study concluded that while planning for health education dissemination in an educated group a focused programme should be formulated in small groups preferably in the form of lectures on specific topics, more so while dealing with participants of higher age group having higher educational qualification.

## Background

Health education is a process by which individuals and groups of people learn to behave in a manner conducive to the promotion, maintenance or restoration of health [1]. Communication in relation to health education involves different modes like lectures, group or panel discussions, symposia, poster or exhibit presentation etc. Every individual mode of health education has its own merits, drawbacks as well as their own sphere of effectiveness. In addition it has to overcome the barriers of communication (e.g. physiological, psychological, environmental and cultural). Research on the effectiveness of different modes of health education dissemination is already in progress to examine the utility of a specific mode of communication in a specific setting [2,3] on a specific group [4]. It has been observed that different educational methods may be specially suitable for different groups of people depending upon their age, sex, educational qualification, background and nature of job [5]. Comparative assessment of effectiveness of different educational methods has also been done on some target groups in different communicational settings [6].

Imparting health education to an educated group is a special arena of interest because of the fact that this educated group may have a major role in the propagation of the achieved knowledge in future. This why communication of health education in a gathering of educated people (e.g. conferences) should have separate specifications in relation to its content and mode of communication. Naturally, this becomes an arena and area of special interest and not much of research is undertaken in this aspect till date. In this backdrop this cross-sectional study was initiated during a scientific conference for making a comparative assessment of different methods of dissemination of health education among educated people.

## Methods

This study was conducted in a scientific conference where 2250 scientists from different branches of science gathered. It was a mega event having health as the theme and experts of various fields attended this conference from different parts of the globe. *This conference was organized by one of the scientific bodies of the country and this conference aimed at disseminating health related issues among the scientists, science managers, policy makers, students and general public. This conference is an annual event (largest scientific gathering of the country), which undertakes an issue every year as the theme and communicates the messages on the theme. This activity being the oldest of the country also is well known for its impact on building awareness and opinion among the scientific community as well as general public. In this way it has not only generated scientific movement in the past involving common mass also but also many times it has substantially influenced policy making*. Various aspects of health promotion, health technology, implication of health in nation's development etc. were discussed and three modes of education dissemination were used; lecture, symposium and exhibition. The conference activity had two divisions; main program and sectional program (fourteen sections were there). *Main programme consisted of deliberation containing discussion on different aspects of science and health, addressing the conference participants at large whereas sectional programmes dealt with a section of the attendees and focused only the issues related to the specific section*. Main program consisted of lectures, symposia and an exhibition whereas the sectional program consisted of lectures and symposia. In the main program, lectures and symposia were of two types; some were based on specific topics and some were based on relatively broader topics. *So far as the lectures are concerned, specific topic lectures included presentation like "cholera-epidemiology, genetics and vaccine development", "role of a tool box of diagnosis for tuberculosis in endemic country", "disease elimination: the kala-azar experience" etc. and broader topic lectures were like "health science and our future", "role of public health in national economics" etc. In case of symposia, the specific topic symposia dealt with the issues like "challenges in combating malaria", "high altitude dysfunction" etc. and the broader topic symposia were on the topics like "environment & health", "bridging the gap between health science and society". Each of these symposia consisted of three or more deliberations from different speakers talking on different aspects of the topic. For example the symposium on "combating malaria" contained topics like "control of malaria in mosquito vector", "current status and strategies for old and new drugs for treatment, prevention and control", "prospects of vaccine", "cost and benefit of malaria control", and "malaria research, development and control strategies"*. Topics of lectures and symposia of sectional programs were specific to the concerned section. *For example, lectures of medical science section consisted of lectures like "factors other than iodine deficiency in endemic goiter", "mechanism of action of enterotoxin of vibrio cholerae", etc. and lectures of environmental science section contained lectures like "biomonitoring of health effects of urban air pollution", "arsenic exposure and effects on liver", etc. Similarly, symposia of medical science section had topic like "development of ergonomics in India" and symposia of environmental science included topics like "environmental endocrine disruptors and reproductive health". These symposia again consisted of different speakers' deliberations on various aspects of the topic of the symposia*. The exhibition contained two types of materials: posters and exhibits. *Posters were prepared on the topics like "prevention of dust related diseases", "how to combat diarrhoeal diseases?" etc. Exhibits were models/instruments, which were displayed and demonstrated for easy conveyance of the related messages. Exhibits contained "spirometer – an instrument early diagnosis of morbidity related to dust related diseases", "model showing transmission of malaria from mosquito vector to human host" etc*.

This cross-sectional interviewer administered questionnaire survey was conducted during the last session of this five-day conference when the opinion of the delegates regarding different programs was asked for. *Necessary ethical clearance was obtained from the institutional ethics committee of National Institute of Occupational Health, India for the purpose of this study*. While calculating the sample size for this study we presumed the lowest choice prevalence to be 10% (as there was no available literature of this nature) and accordingly we calculated the sample size for prevalence study using acceptable range 5–15%. Thus the minimum sample size for 5% level of significance was calculated as 130. We set our target as 150 subjects. Selection of subjects was done by using random numbers generated by Microsoft Excel Software. Initially 3 sections (out of 14 sections) were selected randomly and 50 participants from each section were approached for the study. Of the 150 persons approached for study, 142 agreed to participate.

All the participants were enquired about their choices in relation to all the different aspects of the conference. Analysis of the collected information was undertaken using SPSS release 6.1.4 software. Along with descriptive analysis of the data, univariate analysis was done initially. Afterwards logistic regression technique was applied to obtain contribution of different factors in the choices of the participants. *As we intended to identify the most suitable mode of communication for each division/section of this conference (e.g. lectures, symposia), it was essential to ensure that the findings should be on the basis of merits/demerits of the mode of communication only. For this reason, while going for multivariate analysis, our intention was to observe whether the decision of choices made by the study participants was independent of the factors that might affect the choices (e.g. age, qualification, background, presence of physical barriers of communication, coherence among speakers, etc.)*. Variables like higher qualification (Ph.D/MD or higher), higher designation (Associate Professor or equivalent and above), attending alone or with friends, education background (medical/non-medical) problem in understanding English, origin (urban/rural), noise-congestion-invisibility (absence/presence), coherence among speakers in case of symposia (absent/present) were taken as categorical variables & age was introduced as continuous variable in the logistic regression model. *These variables were introduced as covariates in the logistic regression model and the choices of the study participants (e.g. section programme better, lecture, better, specific topic better etc.) were introduced one by one as the outcome variable. In this way the role of the possible interfering factors on each of the choices of the participants could be evaluated*. In our analysis we accommodated all variables together in the logistic regression model to obtain the contribution of every individual variable adjusting for the effects of other variables.

## Results

Mean age of the study subjects was 33.2 (11.1) years. 67.6% of the subjects were males and 32.4% of the participants were females. 25.4% subjects were more than 40 years of age. 52 (36.6) subjects had higher qualification whereas 44 (31) subjects had higher designation. 16.9% subjects were attending alone whereas rest were along with their friends. Only 16 (11.3) persons had some difficulty in understanding communication in English language. Medical background was found in 8 (5.6) subjects and 20 (14.1) subjects had their origin in rural areas. 54.9% participants reported presence of noise-congestion-invisibility and 69% talked about lack of coherence among the speakers of the symposia.

So far as choice of the participants is concerned, 86 (60.0) subjects opined that sectional programme was better than the main programme. When assessment of main programme was asked for 62% subjects remarked that lectures were best, whereas 29.2% and 13% participants were of the opinion that exhibition and symposium was best. Regarding the sectional programme, it was observed that 65.7% subjects liked lectures rather than symposia. In case of lectures and symposia of main programme, 96 (67.6) and 82 (57.7) subjects respectively liked specific topics better. In the exhibition, exhibits seemed to be more attractive (62%) than the posters (Table-1).

**Table 1:** Opinion of the study subjects regarding effectiveness of communication.

**Assessment area**	**Criteria**	**Number (Percentage)**	**Significance**
Main/Sectional Programme	Sectional Programme was better	86(60.6)	χ^2 ^= 12.68; p < 0.001
	Main Programme was better	56(39.4)	

Main Programme	Lecture was best	85(59.9)	χ^2 ^= 70.46; p < 0.001
	Symposium was best	17(11.9)	-
	Exhibition was best	40(28.2)	χ^2 ^= 10.78; p < 0.01

Sectional Programme	Lecture was better	92(65.7)	χ^2 ^= 27.66; p < 0.001
	Symposium was better	48(34.3)	

Lectures of Main Programme	Specific topic was better	96(67.6)	χ^2 ^= 35.21; p < 0.001
	Broad topic was better	46(32.4)	

Symposia of Main Programme	Specific topic was better	82(57.7)	χ^2 ^= 6.82; p < 0.01
	Broad topic was better	60(42.3)	

Exhibition	Exhibits were better	88(62.0)	χ^2 ^= 16.28; p < 0.001
	Posters were better	54(38.0)	

Table-2 and Table-3, shows the contribution of different factors in determining the choices of the participants. Age of the participants had significant effect in their choices in relation to assessment of lectures and symposia of main programme (multivariate analysis). In case of both lectures and symposia of main programme, significantly positive regression co-efficient showed that specific topic was better for advanced age people. Higher qualification was a significant contributor in preferring sectional programme as such and also in preferring symposia of the sectional programme rather than the lecture (univariate analysis). On multivariate analysis, it was found that higher qualification was a stronger (odds ratio raised from 3.2 to 9.1) contributor for preference of sectional programme. But in case of preference of symposia of sectional programme it became a weaker contributor (though odds ratio increased from 2.9 to 3.7, it became non-significant). On this analysis, higher qualification was also observed to be a significant contributor in case of preference of symposia of main programme. Medical background could not show any significant effect in case of any of the choices except for preference of exhibits (odds ratio was 6.4 in univariate analysis and 19.7 in multivariate analysis) even though the content of all the communications were health related issues. Absence of barriers like noise-congestion-invisibility was a significant contributor (multivariate analysis) while preferring sectional programme as such and also for preference of exhibits. Coherence among the speakers appeared to be the most important factor while assessing symposia of both main and sectional programme (univariate analysis). The significance of this factor increased many folds when the data was subjected to multivariate analysis.

**Table 2:** Distribution of odds ratio in relation to different contributing factors (univariate analysis)

Covariates	Choice of Main/ Sectional Programme (Sectional better)	Assessment of Main Programme (Symposium Better)	Assessment of Sectional Prog. (Symposium better)	Assessment of Exhibition (Exhibit better)
Higher Qualification	3.19 (1.38 – 7.45)	2.5 NS	2.92 (1.27 – 6.82)	1.13 NS
Higher Designation	2.17 NS	1.17 NS	2.77 (1.15 – 6.82)	1.27 NS
Medical Background	0.2 NS	_____	1.9 NS	6.43 (1.09 – 48.98)
Coherence of the Speakers in Symposium	NA	32.0 (6.28 – 220.10)	43.0 (13.45 – 146.12)	NA

NS = Non Significant, NA = Not Applicable, Figures within parenthesis indicate 95% Confidence Interval.

Covariates like age>40, attending alone, problem of understanding English, urban background, absence of physical barriers like noise-congestion-invisibility did not show any significant impact on the choices of the study participants. Choices like lecture in main programme better, exhibition in main programme better, lecture in sectional programme better, specific topic in main programme symposium better and specific topic in main programme lecture better were independent of all the covariates.

**Table 3:** Distribution of odds ratio in relation to different contributing factors (multivariate analysis)

Covariates Choice of Main/	Sectional Programme (Sectional better)	Assessment of Main Programme Symposium better	Assessment of Main Prog. Symposium (Specific topic better)	Assessment of Main Prog. Lecture (Specific topic better)	Assessment of Sectional Prog. (Symposium better)	Assessment of Exhibition (Exhibit better)
Age	NS	NS	p < 0.001, Reg. Co-eff. = 0.1842	p < 0.001, Reg. Co-eff. = 0.1641	NS	NS

Higher Qualification	9.1 (1.20–17.01)	40.62 (14.12 – 67.12)	1.57 NS	0.67 NS	3.71 NS	0.65 NS
Medical Background	0.1 NS	0.06 NS	0.32 NS	___	2.82 NS	19.75 (10.34 – 29.16)
Absence of Noise, Congestion, Invisibility	3.46 (1.09–5.82)	0.02 NS	0.51 NS	0.47 NS	0.57 NS	7.78 (2.36 – 13.21)
Coherence of the Speakers in Symposium	NA	105.79 (95.91–115.68)	NA	NA	308.77 (301.39 – 316.14)	NA

NS = Non Significant, NA = Not Applicable, Figures within parenthesis indicate 95% Confidence Interval.

Covariates like higher designation, attending alone, problem of understanding English and urban background did not show any significant impact on the choices of the study subjects. Choices like lecture in main programme better, exhibition in main programme better and lecture in sectional programme better were independent of all the covariates.

## Discussion

Sectional programmes were being attended by concerned audience in the form of a relatively smaller group and the topics were specific to the concerned section. This may have been the reason of participants' preference towards sectional programme over main programme (main programme was addressing a broader audience of non-specific nature). In main as well as sectional programmes, lectures were preferred over symposia. This may be due to the fact that educated mass may have liked a comprehensive communication by a single deliverer more than a non-coherent message from multiple communicators (69% of subjects reported that there was poor coherence among the speakers of the symposia). *For example a comprehensive lecture on "cholera – epidemiology, genetics and vaccine development" by a single deliverer has been more acceptable and useful than a symposium on "challenges in combating malaria" where different aspects of the topics were dealt with by different experts. This may have been due to the fact that the audience have liked a focused discussion a limited topic rather than a composite message on different aspects of a relatively larger area at a time. Lack of linkage between the speakers may also have been a matter of concern because it hinders the process of comprehensive learning on a larger topic*. In case of main programme lecture had more impact than exhibition even. *This has probably been a special feature of the educated audience. In spite of the lucidity of the message delivery inherent in exhibition, the study participants have opted more for lectures possibly because of the reason that the lectures contained optimum volume of messages delivered in a more elaborate and systematic manner*. The completeness of a topic achieved through a lecture may have been the more attraction than the discreteness of message passed though individual posters or exhibits. Though people from a varied discipline of science were the audience in the main programme, specific topics were better in the lectures as well as in the symposia. *This observation has been a salient finding of this study. Specific topics have been preferred everywhere by educated audience over relatively broader topics. People may have found specific in depth knowledge on topics like "cholera" or "malaria" more useful rather than general discussion on relatively broader topics like "environment and health" or "role of public health in national economics"*. In the exhibition, exhibits have carried more impression than the posters. *Participants may have liked hands on experience of operating different exhibits (instruments) more than the message disseminated by the posters*.

So far as different possible determining factors of participants' decision are concerned, higher qualification has been a contributing factor in participants' preference towards sectional programme (OR 9.1, 95% CI 1.2–17.0) and also in choosing main programme symposia better (OR 40.6, 95% CI 14.1–67.1). In the preference of symposia of sectional programme also higher qualification played a role. In this case, higher designation also showed some impact (though significant in univariate analysis it was not significant in multivariate analysis). Thus, qualification (in some cases designation also) has emerged to be a decisive factor in peoples' choice towards more specific subject oriented programme (sectional programme) and also in their affinity to symposia. Increased age was a significant contributor in participants' preference towards specific topics. Medical background has helped people only in understanding exhibits. The scientific details may have been easily understandable to such people due to their medical background. Physical barriers of communication (noise-congestion-invisibility) have contributed significantly in subjects' preference towards sectional programme as such and also in the choice of exhibits rather than the posters. *This finding points towards the fact that physical barriers of communication play an important role in the success of a health education dissemination programme*.

Some of the earlier studies have already stressed the need of exploding the background and character of the recipient group while imparting health education [7]. Some studies have shown the success of different modes of communication in different situations [8-12]. View of different recipient groups are different towards various modes of communication and the success of a health education programme depends on the planning of the structure of such a programme taking care of all the relevant factors [13]. The implication of a well-planned health education programme is far spreading and such a programme has a great potential in changing public attitudes [14,15]. *This study also has strengthened the idea of planning the health education programme according to the background and character of participating groups. While addressing the issue of imparting health education to an educated mass, this study has come out with very specific observations. It has showed that health education programme in the form of lectures on specific topics dealing with a small section is more likely to succeed in case of educated audience. This study has pointed out that if an exhibition is planned for such audience, it should contain more and more exhibits rather than posters. Moreover, it has also been observed in this study that higher age has a positive role in participants' choice towards specific topics. Higher qualification has some positive impact in choice towards focused programme involving smaller groups. Importance of basic criteria for the success of a heath education endeavor like comprehensiveness of the content, role of physical barriers of communication and coherence among multiple speakers covering various aspects of a topic has also been highlighted by virtue of this study*.

## Conclusion

This study has come out with an important but relatively less lighted aspect of health education dissemination. It has addressed some of the important issues in imparting health education to a gathering of educated people, representing different fields of knowledge. On one hand this study has spoken for preference of a well-designed comprehensive lecture rather than a non-coherent symposia while on the other hand it has stressed the need of adoption of specific topics (more so with increasing age of the receptor population). *At the end, this study has concluded that while planning for health education dissemination in an educated group a focused programme should be formulated in small groups preferably in the form of lectures on specific topics, more so while dealing with participants of higher age group having higher educational qualification*.

## Competing interests

The author(s) declare that they have no competing interests.

## Authors' contributions

***AS: ***Planned and designed the study, executed the study, analyzed the data and prepared the final write up.

***EP: ***Executed the study, associated with the planning of the study, contributed in write up and also in literature search.

***MM: ***Executed the study, helped in data analysis, contributed in literature search and write up.

## Pre-publication history

The pre-publication history for this paper can be accessed here:


